# Retinal Vascularity in Military Pilots in Relation to the Type of Aircraft, Flight Altitude and Total Hours of Flight

**DOI:** 10.3390/jcm14082671

**Published:** 2025-04-14

**Authors:** Justyna Mędrzycka, Grzegorz Rotuski, Radosław Różycki, Joanna Gołębiewska

**Affiliations:** Department of Ophthalmology, Military Institute of Aviation Medicine, 01-755 Warsaw, Poland; jmedrzycka@wiml.waw.pl (J.M.); rrozycki@wiml.waw.pl (R.R.); jgolebiewska@wiml.waw.pl (J.G.)

**Keywords:** military pilots, aviation, high-altitude retinopathy, OCT angiography, retinal microvasculature, retinal vessel density

## Abstract

**Background**: The aim of the study was to assess retinal circulation in military pilots, as well as to determine the relationship between the type of aircraft, flight altitude, total hours of flight time and parameters of retinal circulation, using OCT angiography (OCT-A). **Methods**: This cross-sectional study enrolled 44 military pilots and 44 controls. The inclusion criteria encompassed healthy adult men. Due to the fact that military pilots cannot suffer from any vision defects or any other eye disease, the exclusion criteria concerned the control group and included refractive error exceeding −3 diopters (D) and +3 D and concomitant eye diseases, such as any retinal or choroidal pathologies, glaucoma, uveitis. The exclusion criteria for both groups were low-quality OCT-A images. Subsequently, the results of the measurements obtained for 176 eyes were included in further descriptive and multivariate analyses, of which 88 were in the pilot group versus 88 in the comparison group. **Results**: The total vessel density in superficial and deep capillary plexuses was significantly decreased (*p* = 0.0176, *p* < 0.0001, resp.) the longer the flight experience, particularly in the parafoveal region (*p* = 0.0299 and *p* < 0.0001, resp.). Moreover, the foveal avascular zone area was significantly increased proportionally to the total hours of flight (*p* = 0.0083). Also, the total vessel density was increased with a higher flight altitude in the deep capillary plexus (*p* = 0.0042), especially in the parafoveal region (*p* = 0.0110). **Conclusions**: Gravitational forces manifesting in the unique conditions of the flight of military pilots seem to induce microvascular changes in the retina.

## 1. Introduction

Staying at high altitudes above sea level is associated with systemic hypoxia due to the drop in atmospheric pressure and thinning of the air [1]. For the human body, this is an unfavorable phenomenon associated with the risk of high-altitude sickness syndrome, which includes such clinical entities as acute mountain sickness (AMS), high-altitude pulmonary edema (HAPE), high-altitude cerebral edema (HACE) and high-altitude retinopathy (HAR). Typically, the first symptoms begin to appear at an altitude above 2500 m above sea level, where the availability of oxygen in the air becomes insufficient to maintain proper functioning of the body, but this depends on the individual’s adaptation mechanisms and overall health [1,2]. High-altitude retinopathy (HAR) was first described in 1969 [3]. It may manifest through the increased diameter and tortuosity of retinal vessels [4], hyperemia of the optic disc, diffuse or pinpoint preretinal hemorrhages that occur mainly peripherally and hemorrhages into the vitreous body [3,5]. The exact pathogenesis of HAR is not fully understood. Hypoxia essential disturbs cellular metabolism, which is mostly damaging to cardiomyocytes and neuronal cells, having the highest energetic demand in the body. Cellular stress can trigger an inflammatory reaction leading to increased vascular permeability that can cause retinal tissue edema or hemorrhages. Treatment consists of descending to a lower altitude, oxygen therapy and hydration; also, corticosteroids can be considered in severe cases.

Good visual acuity depends, among other things, on blood flow in the retina, optic nerve and choroid. These are regulated by the oxygen pressure in the tissues, and a decrease in the partial pressure of oxygen in arterial blood causes an increase in blood flow [6]. In ischemic states, compensatory mechanisms always lead to maintaining circulation in the posterior pole—in the macula. It has been observed that at an altitude of 4000–6000 m, as a result of hypoxia, the retinal arteries dilate [7]. Flying at an altitude of over 7000 m leads to the breakdown of these mechanisms. In order to prevent hypoxia during high-altitude flights, various types of oxygen devices are used, which operate by enriching the inhaled air with oxygen. These devices are able to maintain the appropriate oxygen saturation in the inhaled air up to an altitude of 12,000 m. After exceeding this altitude, the atmospheric pressure is lower than the partial pressure of oxygen necessary for proper gas exchange in the lungs and tissues. This involves the need to administer oxygen at a pressure higher than that prevailing in the surrounding atmosphere. In addition to the oxygen device, the pilot must then be equipped with compensatory clothing and an oxygen mask that firmly fits the face or a tight helmet. In the case of fighter pilots, a pressurized cabin and compensating clothing are used as emergency protection in the event of sudden cabin depressurization [8]. This in turn can lead to decompression retinopathy. Altitude-related decompression illness predominantly occurs during airplane flights that are not pressurized or following pressurization system malfunctions. A higher risk of developing this condition concerns flights at altitudes exceeding 18,000 feet within a brief timeframe, rapid ascents, prolonged periods spent at higher altitudes, older age, dehydration or being under the influence of alcohol [9].

The aim of the study was to assess the retinal circulation in military pilots, as well as to determine the relationship between the type of aircraft, flight altitude, total hours of flight time and parameters of retinal circulation, using non-invasive optical coherence tomography angiography (OCT-A).

## 2. Materials and Methods

This cross-sectional study was conducted between September 2022 and March 2023. The study was approved by the Ethics Committee of the Military Institute of Aviation Medicine in Warsaw, Poland and followed the tenets of the Declaration of Helsinki. The participants signed a written consent form before enrolling in the study. The study group consisted of military pilots from 4 military bases across the country. Controls were recruited from male ground staff in these bases. The inclusion criteria encompassed healthy adult men (≥18 years old). Due to the fact that military pilots cannot suffer from any vision defects or any other eye disease, the exclusion criteria concerned the control group and included refractive error exceeding −3 diopters (D) and +3 D and concomitant eye diseases, such as any retinal or choroidal pathologies, glaucoma, uveitis. The best-corrected visual acuity (BCVA) of controls was 20/20. Exclusion criteria for both groups were low-quality OCT-A images. Each participant in the study underwent a detailed and thorough ophthalmological examination of both eyes. Subsequently, the results of measurements obtained for 176 eyes were included in further descriptive and multivariate analyses, of which 88 were in the pilot group versus 88 in the comparison group.

Spectral domain OCT (SD-OCT) and OCT angiography scans were performed on all subjects using RTVue XR Avanti with AngioVue software, regulation number 21 (Optovue, Fremont, CA, USA), taking 3 mm × 3 mm images of the macula centered on the foveola, in high-resolution mode. In this technology, tissues are visualized through interferometry, where retinal layers reflect the infrared beam distinctly, which enables it to form a detailed map of the examined structure. Each OCT-A en face image contains 304 × 304 pixels created from the intersection of 304 vertical and 304 horizontal B-scans [10]. AngioVue automatically segments the area into four layers, including superficial capillary plexus (SCP), deep capillary plexus (DCP), outer retina layer and choriocapillaris. The SCP en face image was segmented with an inner boundary at 3 µm beneath the internal limiting membrane and an outer boundary set at 15 µm beneath the inner plexiform layer, whereas the deep capillary plexus en face image was segmented with an inner boundary 15 µm beneath the inner plexiform layer and an outer boundary at 70 µm beneath the inner plexiform layer. Integrated automated algorithms provided by the machine’s software were used to quantify the foveal avascular zone (FAZ) area (mm^2^) and macular vascular density (%). FAZ area was automatically calculated for superficial plexus. Capillary vascular density in macular and paramacular region were measured both in superficial and deep plexuses. Vessel density is calculated as the percentage area occupied by flowing blood vessels in the selecting region, which enables quantitative assessment of microvasculature. The foveal region is a 1 mm circle, while the parafoveal region is a ring with an internal diameter of 1 mm and external diameter of 3 mm. Foveal thickness (FT) (µm) and parafoveal thickness (PFT) (µm) data were obtained from retinal maps, using the same device. Poor-quality scans (quality < 6), with motion artefacts or blurred images, were excluded. The data collected from both eyes of the studied patients were taken into analysis.

Numerical traits were described using measures of central tendency—weighted arithmetic mean and median—along with measures of dispersion—standard deviation, lower and upper quartile and minimum and maximum values. Initially, the Shapiro–Wilk W test was carried out to assess the normality of the distribution and Levene’s test was fitted to assess the homogeneity of variances. A multivariate analysis of variance (ANOVA) without repetitions was performed to assess the statistical significance of differences between the two study groups in the values measured, controlling for their age. When dealing with non-normally distributed variables, generalized linear models (GLMs) with robust standard errors to outliers were used. In the above statistical models, intra-subject variability correction was applied due to the fact that all study participants underwent ophthalmological assessments and measurements in both eyes. In order to assess the strength, direction and statistical significance of possible correlations of selected numerical variables, the Spearman’s rank correlation ρ coefficient was calculated. A level of *p* < 0.05 was considered statistically significant. All of the statistical calculations were performed using Statistica™, release 14 (TIBCO Software Inc., Palo Alto, CA, USA).

## 3. Results

A total of 88 eyes of 44 military pilots were included in this study and compared to the OCT and OCT-A data of 88 eyes of 44 men who were ground staff. The average age of the pilots was 39.7 years (SD = 6.9 years; range: 27–50 years), while the average age of the control group was 41.2 years (SD = 6.3 years; range: 28–53 years). The mean ages of both groups did not differ significantly (*p* = 0.2771). The examined pilots were flying supersonic fighter aircraft (F-16, MiG-29), helicopters (Mi-8, W-3), tactical airlifters (C-130) and narrow-body airliners (Boeing 707 and 737) (Table 1). They were split into groups based on their total flight time and flight altitude.

Vessel density in the SCP and DCP (*p* = 0.9002 and *p* = 0.2383, respectively), vessel density in the foveal (*p* = 0.7089 and *p* = 0.7911, resp.) and parafoveal region (*p* = 0.5157 and *p* = 0.1637, resp.), the total radial peripapillary capillary density (*p* = 0.4109) and the density in the optic disc region (*p* = 0.2283) did not differ significantly between both groups (Table 2). The FAZ area was comparable in the two groups (*p* = 0.7940).

By extracting pilot groups based on their total time of flight, statistically significant results were obtained regarding the total vessel density in the SCP and DCP (*p* = 0.0176, *p* < 0.0001, resp.), showing increasingly reduced values the longer the flight experience (Table 3). This was mainly expressed in the parafoveal region (*p* = 0.0299 and *p* < 0.0001, resp.), with no statistical significance in the fovea. Moreover, a very significant correlation was observed with the FAZ area, which increases proportionally to the number of hours spent mid-air in the aircraft (*p* = 0.0083).

On changing the grouping criteria to the typical altitude of flight of the examined pilots, a very significant difference was noted in the total vessel density in the DCP (*p* = 0.0042) in the parafoveal region (*p* = 0.0110) without statistical significance in the fovea (*p* = 0.5982) (Table 4). In this case, the FAZ area did not differ significantly between the groups. The only significant difference at the cut-off of 10,000 m was found in the radial peripapillary capillary density, showing a decrease in this parameter (*p* = 0.0005).

## 4. Discussion

To the best of our knowledge, this is the first study of retinal microcirculation in military pilots this detailed. Based on these results, we can assume that these unique working conditions cause a depletion in retinal microvasculature. Particularly predisposed was the perifoveal region, concerning the deep capillary plexus of the retina. A tendency of abnormalities happening predominantly in the DCP was also observed in other vasculopathies like scleroderma [11]. This might be due to glial cells, particularly astrocytes, located most densely in the perifoveal region [12]. Perifoveal capillary branching and density loss are also described in patients with diabetes mellitus [13]. Low retinal microvascular density and vascular branching complexity correlates with a higher risk of mortality and cardiometabolic disease [14]. As a result of the subclinical inflammation process, local hyperemia can occur, leading, through repetitive events, to tissue remodeling.

Another study on individuals exposed to high altitudes showed dilated retinal vessels in the SCP and constricted ones in the DCP; this difference in vascular wall reactivity in both layers might explain why we observed decreased vessel density, particularly in the DCP, in the subgroups of pilots with more flight experience and higher altitudes of flight [15]. It is likely also that atrophic changes happened in the retina due to unfavorable conditions preventing stable blood flow and surpassing the amplitude of changes overpassing autoregulation mechanisms, leading to the decimation of blood vessels. This is proven by the enlargement of the FAZ area proportional to the total time spent in flight. FAZ enlargement is also seen in diabetic retinopathy, leading us to believe the changes we observe in military pilots come from ischemic events [16]. A significant FAZ area increase was associated with moderately faster rates of GCC thinning, but not a macular vessel density change in eyes with glaucoma [17]. The fact that we have not noted a similar deficit in the fovea itself might be explained by vascular remodeling in order to keep the best nutrition for the area of central vision.

A meta-analysis showed an increase in vascular density related to exercise, particularly in the deep plexus [18]. The heightened cardiac output in cardiorespiratory or endurance sports induces both arteriole and venule diameter growth. Physical stamina is necessary in military pilots, who experience visual field narrowing mostly related to centrifugal force, and have to perform leg- and abdominal-muscle-pumping maneuvers in order to reestablish blood flow to the brain and orbits. This may explain why the net vascular anastomoses in military pilots flying at greater heights are reorganized comparably to changes seen after short physical exercise.

The mechanism of these alterations appears to be rather vascular than mechanical, as peripheral retinal degenerations had a similar incidence in military pilots to that of the general population [19]. Even with a similar incidence, prophylactic laser photocoagulation is indicated in jet aircraft pilots, especially when they are present in the upper temporal quadrant of the retina due to higher risk of retinal detachment. Aircraft type was also identified as a risk factor, with military pilots having a higher risk than carrier and helicopter pilots [20].

Several cases of flight-associated HAR [21,22,23] have been described, likely attributable to cabin pressure fluctuations. In general, it affects mainly unacclimatized individuals exposed to hypobaric hypoxia. Other manifestations of HAR include venous and arterial dilatation, intraocular or intraorbital hemorrhages, peripapillary hyperemia or papilledema and, ultimately, cystoid macular edema. Risk factors include higher altitude and longer exposure, increased blood viscosity, lower SaO_2_ and higher baseline intraocular pressure (IOP) [24]. The most widely accepted theory is a hypoxia-induced autoregulatory mechanism in the retinal vasculature. Deficiency in oxygen levels at high altitudes leads to hypoxia, which induces various compensatory mechanisms in retinal circulation, sometimes resulting in retinal hemorrhages. Variation in hematocrit levels and an increase in venous pressure due to raised intracranial pressure are other theories. A study on acute mountain sickness (AMS) revealed an increased retinal radial peripapillary capillary flow density, predominantly in the nasal quadrant, and concluded that overperfusion of the microvascular bed was the main finding associated with developing AMS symptoms in the examined individuals [25].

At high altitudes, both retinal arterioles and venules increased in diameter, the latter slightly more, enhancing vascular perfusion. This results from lower partial oxygen pressure (PaO_2_) and a high demand of photoreceptors in the oxygen supply [24]. Frequent changes in blood flow through the retinal barrier might cause depletion of retinal cells, but could also be the cause of retinal pigment epitheliopathy, being the underlying issue of central serous chorioretinopathy, though the latter is poorly explored in military pilots and might be related to stressful working conditions. The same goes for non-arteritic ischemic optic neuropathy [26]. Disturbances in retinal function are also shown on electrophysiological examinations; for instance, the S-cone b-wave decreased significantly during rapid ascent, as seen using electroretinography. Lower perfusion followed by blood overflow is detrimental to neurons and is only partially mitigated by autoregulation mechanisms.

Total vessel density (TVD) showed a direct relationship with geographic altitude [27]. In a study comparing individuals with normal erythrocytes against those with polycythemia in a population living at high altitudes, the latter had significantly lower TVDs and significantly higher subfoveal choroidal thicknesses, while the FAZ area was comparable between the two groups [28]. Short-term exposure to high altitudes induces retinal microcirculation disturbances and autoregulatory responses in healthy individuals, which is probably attributed to arterial PaO_2_ and endothelial dysfunction under hypoxic conditions. Endothelial dysfunction in arterioles is more susceptible to hypoxia than that in venules, and the reaction is almost immediate [29]. Elevated levels of oxidative stress can induce mitochondrial damage, an inflammatory response and apoptosis, ultimately leading to structural and functional changes of the retina [30].

To delve even deeper into understanding microcirculation alterations in military pilots, another important entity to consider in this aspect is the spaceflight-associated neuro-ocular syndrome (SANS), which is currently under multiple investigations worldwide [31]. The typically observed changes are posterior globe flattening along with hyperopic shift; choroidal-congestion-inducing retinal folds, resulting most probably from cephalad fluid accumulation in the upper body; and optic disc swelling with probable ischemic changes seen as soft exudates in the retina [32]. Some of these changes are similar to the pathology observed in intracranial hypertension, but without chronic headaches or tinnitus, a posterior displacement of Bruch’s membrane and a pattern of obesity in SANS.

A study of changes occurring in parabolic flights described that microgravity causes retinal venous dilation and arterial constriction, related to an IOP increase; this is in opposition to hypergravity, where the arteries become more dilated and veins narrowed, while the IOP is reduced. A correlation was found with diastolic blood pressure—decreased in microgravity and increased during hypergravity, with no alterations of systolic blood pressure. Although the ocular perfusion pressure results from the MAP—IOP equation, where MAP stands for mean ocular pressure [33], we need to additionally consider the gravitational component in military pilots’ flight routines that is further disrupting retinal microcirculation.

The limitations of this study comprise a relatively small group of participants and low representativeness of the sample—all subjects were Caucasian and the lack of ethnic diversity in this group may not reflect all military pilots across the world. Also, the study included only men, due to no female military pilots working in either of the military bases at the time.

## 5. Conclusions

Military pilots in jet aircrafts experience subclinical retinal microvasculature alterations due to challenging environmental conditions relative to the Earth’s surface. Due to unusual forces related to acceleration, lower partial oxygen pressure at higher altitudes and rapid barometric changes, these perturbations can prove harmful to the metabolically demanding retina, particularly due to the lack of time for adaptation in such fast-changing conditions. Non–invasive OCT-A seems to be a very promising tool to diagnose and monitor retinal changes in military pilots. This research can shed light on the understanding of retinal pathology, though a study on a larger cohort would be advised to obtain more conclusive results and strengthen the implications for aviation professionals.

## Figures and Tables

**Table 1:** Aircraft examined pilots flew, their typical flight altitude and estimated time of flight.

Type of Aircraft	Typical Flight Altitude (m.a.s.l. ^1^)	Total Hours of Flight (hours)	Age of Each Pilot (Year)
Mi-8 (Helicopter)	<5000	<1000	38
		2000–3000	45,48,49
		3000–4000	46,49
W-3 (Falcon)	5000–10,000	1000–2000	35,37,40,40
		2000–3000	42,43,46
		3000–4000	41,45,50
C-130 (Hercules)	5000–10,000	2000–3000	46
Boeing 737	10,000–15,000	1000–2000	47
Boeing 707	10,000–15,000	2000–3000	43
MiG-29	15,000–20,000	<1000	34
		1000–2000	42,49
F-16	15,000–20,000	<1000	27,28,29,30,31
		1000–2000	29,31,32,33,34,35,39
		2000–3000	37,38,40,47,48,49
		3000–4000	49

^1^ m above sea level.

**Table 2:** Selected parameters measured in military pilots vs control group.

Analyzed Trait	Study Group	Statistical Parameter					Statistical Significance Level (*p*)
		M	SD	Me	Q1–Q3	Min.–Max.	
tSVD	Pilots	48.4	2.5	48.8	47.4–50.0	39.2–53.5	0.9002
	Controls	48.3	2.7	48.9	46.8–49.8	40.2–55.6	
tDVD	Pilots	54.1	2.6	54.5	53.0–55.8	44.3–58.8	0.2383
	Controls	53.4	2.9	53.7	51.6–55.7	43.0–59.3	
fSVD	Pilots	22.6	5.3	23.3	19.4–25.4	9.6–51.3	0.7089
	Controls	22.1	6.2	22.0	18.2–25.4	8.2–41.0	
fDVD	Pilots	38.3	5.5	38.9	34.7–42.3	25.0–50.4	0.7911
	Controls	37.9	6.6	37.7	34.4–42.6	20.7–53.1	
pSVD	Pilots	51.1	2.6	51.4	50.0–52.8	41.8–56.3	0.5157
	Controls	50.6	4.0	51.6	48.9–52.9	23.8–57.4	
pDVD	Pilots	55.4	2.5	55.5	54.2–57.0	46.9–59.8	0.1637
	Controls	54.7	2.8	54.9	53.2–57.0	44.4–60.9	
PERIM	Pilots	1.850	0.334	1.861	1.608–2.029	0.942–2.603	0.5703
	Controls	1.892	0.376	1.925	1.580–2.140	0.691–2.845	
FAZ	Pilots	0.233	0.163	0.221	0.158–0.284	0.060–1.570	0.7940
	Controls	0.229	0.089	0.219	0.164–0.285	0.033–0.517	
TRCD	Pilots	49.7	1.9	49.7	48.3–51.0	44.4–53.4	0.4109
	Controls	49.9	1.7	49.8	48.8–51.2	45.1–56.0	
ORCD	Pilots	53.9	4.0	54.1	51.5–56.9	41.4–62.2	0.2283
	Controls	54.5	4.1	55.1	53.0–56.2	42.1–63.5	
PRCD	Pilots	51.7	2.2	51.9	50.2–53.3	44.6–56.1	0.9456
	Controls	51.8	2.2	51.6	50.4–53.1	46.8–57.3	

M = mean, SD = standard deviation, Me = median, Q1–Q3 = first to third quartile, tSVD = total superficial vessel density [%], tDVD = total deep vessel density [%], fSVD = foveal superficial vessel density [%], fDVD = foveal deep vessel density [%], pSVD = perifoveal superficial vessel density [%], pDVD = perifoveal deep vessel density [%], PERIM = perimeter circumference of the foveal avascular zone [mm^2^], FAZ = foveal avascular zone [mm^2^], TRCD = total radial capillary density [%], ORCD = radial capillary density on the optic disc [%], PRCD = radial capillary density around the optic disc [%].

**Table 3:** Selected parameters measured in military pilots based on total hours of flight.

Analyzed Trait	Flight Time (Hours)	Statistical Parameter					Correlation ρ Coefficient and Statistical Significance Level (*p*)
		M	SD	Me	Q1–Q3	Min.–Max.	
tSVD	<1000	50.1	1.5	50.2	49.2–50.6	47.7–53.5	−0.2525 0.0176
	1000–2000	48.0	2.9	48.7	47.2–49.7	30.2–53.3	
	2000–3000	48.8	1.6	48.8	47.6–49.7	45.6–52.5	
	3000–4000	46.9	3.1	46.3	44.6–50.0	41.4–51.5	
tDVD	<1000	55.2	0.7	55.2	54.6–55.7	54.0–56.4	−0.5039 <0.0001
	1000–2000	55.1	2.6	56.0	53.7–57.0	47.4–58.8	
	2000–3000	53.4	1.7	53.7	51.9–54.7	49.2–56.2	
	3000–4000	51.5	3.4	53.0	50.0–53.4	44.3–55.3	
fSVD	<1000	25.7	8.3	23.8	22.4–28.2	16.7–51.3	−0.1669 0.1202
	1000–2000	22.4	4.2	23.1	19.8–25.0	11.7–30.3	
	2000–3000	22.5	3.8	23.6	19.0–25.6	14.8–27.6	
	3000–4000	19.9	5.8	20.4	16.1–24.3	9.6–27.7	
fDVD	<1000	40.0	5.0	40.5	36.8–41.3	32.9–50.4	−0.1853 0.0839
	1000–2000	39.0	5.2	38.5	35.3–42.8	25.7–48.7	
	2000–3000	38.6	5.1	40.2	33.7–42.3	29.1–45.3	
	3000–4000	34.1	6.6	32.8	29.2–39.8	25.0–44.3	
pSVD	<1000	52.7	1.6	52.6	51.6–53.7	50.1–56.3	−0.2316 0.0299
	1000–2000	50.7	3.0	51.2	50.0–52.4	41.8–55.5	
	2000–3000	51.5	1.8	51.4	50.1–52.6	48.0–55.9	
	3000–4000	49.6	3.4	48.9	47.2–53.4	43.0–53.9	
pDVD	<1000	56.2	1.1	56.2	55.3–57.0	54.4–58.1	−0.4698 <0.0001
	1000–2000	56.6	2.5	57.3	54.9–58.4	49.4–59.8	
	2000–3000	54.6	1.6	55.1	53.7–55.7	50.3–57.0	
	3000–4000	53.1	3.2	54.4	51.1–55.3	46.9–56.6	
PERIM	<1000	1.704	0.342	1.757	1.464–1.957	1.153–2.275	0.2643 0.0139
	1000–2000	1.805	0.330	1.827	1.606–2.012	0.942–2.603	
	2000–3000	1.864	0.319	1.902	1.582–2.159	1.378–2.396	
	3000–4000	2.089	0.277	2.033	1.838–2.347	1.771–2.498	
FAZ	<1000	0.186	0.069	0.195	0.124–0.236	0.083–0.313	0.2828 0.0083
	1000–2000	0.249	0.244	0.214	0.157–0.250	0.060–1.570	
	2000–3000	0.212	0.069	0.190	0.154–0.285	0.115–0.316	
	3000–4000	0.284	0.052	0.294	0.233–0.317	0.191–0.367	
TRCD	<1000	50.5	1.8	50.7	49.3–51.7	47.5–53.4	−0.0270 0.8030
	1000–2000	49.2	2.1	49.5	48.0–50.8	44.4–52.6	
	2000–3000	49.5	1.6	49.3	48.6–50.5	46.0–52.4	
	3000–4000	50.3	1.9	50.9	48.9–51.3	46.2–53.0	
ORCD	<1000	55.9	3.3	56.6	52.3–58.6	51.9–60.6	−0.1167 0.2790
	1000–2000	53.8	3.6	54.0	51.1–55.8	47.7–60.8	
	2000–3000	53.3	4.6	53.7	50.9–56.0	41.4–62.2	
	3000–4000	53.6	4.5	55.2	50.6–57.0	45.2–58.1	
PRCD	<1000	51.9	2.1	52.0	50.6–53.6	48.7–54.9	0.1585 0.1403
	1000–2000	51.2	2.5	50.9	49.9–53.1	44.6–56.1	
	2000–3000	51.7	2.0	52.2	50.2–53.1	47.8–55.1	
	3000–4000	53.0	1.6	53.3	51.5–54.5	50.2–55.1	

**Table 4:** Selected parameters measured in military pilots based on flight altitude.

Analyzed Trait	Flight Altitude (m.a.s.l.)	Statistical Parameter					Correlation ρ Coefficient and Statistical Significance Level (*p*)
		M	SD	Me	Q1–Q3	Min.–Max.	
tSVD	<5000	49.1	1.9	49.4	48.0–50.6	44.6–51.5	0.0100 0.9263
	5000–10,000	47.6	3.1	48.5	46.4–49.7	39.2–51.9	
	10,000–20,000	48.7	2.2	48.9	47.6–50.2	44.0–53.5	
tDVD	<5000	53.0	3.3	54.3	51.9–54.8	44.3–56.2	0.3023 0.0042
	5000–10,000	53.1	2.9	53.2	51.8–55.3	45.4–57.8	
	10,000–20,000	54.8	1.9	55.0	53.7–56.2	49.2–58.8	
fSVD	<5000	22.2	4.5	24.1	17.2–25.6	14.8–27.7	0.1046 0.3321
	5000–10,000	21.8	3.4	23.1	19.3–24.0	15.8–28.0	
	10,000–20,000	23.2	6.3	23.6	19.6–26.0	9.6–51.3	
fDVD	<5000	38.1	6.1	41.0	33.3–42.4	26.6–44.3	0.0837 0.5982
	5000–10,000	37.5	4.1	38.1	34.9–40.6	29.8–44.0	
	10,000–20,000	38.9	6.0	40.0	34.7–43.4	25.0–50.4	
pSVD	<5000	51.6	1.9	51.7	50.6–53.0	47.0–53.8	0.0358 0.7404
	5000–10,000	50.4	3.2	51.0	49.2–52.6	41.8–55.1	
	10,000–20,000	51.4	2.4	51.6	50.0–53.1	46.0–56.3	
pDVD	<5000	54.4	3.1	55.4	53.0–56.3	46.9–57.0	0.2699 0.0110
	5000–10,000	54.7	2.9	54.6	53.4–56.6	47.5–59.8	
	10,000–20,000	56.1	2.0	56.2	54.9–57.5	50.3–59.6	
PERIM	<5000	1.931	0.277	1.904	1.728–2.193	1.520–2.318	−0.1481 0.1736
	5000–10,000	1.914	0.298	1.911	1.608–2.005	1.520–2.498	
	10,000–20,000	1.795	0.360	1.781	1.557–2.026	0.942–2.603	
FAZ	<5000	0.234	0.064	0.218	0.179–0.293	0.144–0.316	−0.2118 0.0503
	5000–10,000	0.283	0.270	0.239	0.176–0.296	0.133–1.570	
	10,000–20,000	0.205	0.080	0.196	0.152–0.249	0.060–0.408	
TRCD	<5000	50.8	1.6	51.2	49.2–51.9	48.5–53.0	−0.0528 0.6253
	5000–10,000	49.1	1.8	49.3	48.0–50.4	44.9–52.4	
	10,000–20,000	49.7	1.9	49.7	48.2–51.0	44.4–53.4	
ORCD	<5000	53.8	3.6	53.8	50.9–55.5	48.3–60.6	0.0914 0.3970
	5000–10,000	53.3	3.8	55.0	50.3–56.5	45.2–58.1	
	10,000–20,000	54.3	4.2	54.0	51.9–57.9	41.4–62.2	
PRCD	<5000	53.5	1.2	53.7	52.4–54.4	51.3–55.1	−0.3653 0.0005
	5000–10,000	52.1	1.9	52.4	50.3–53.2	48.7–56.1	
	10,000–20,000	51.1	2.3	51.2	49.8–52.3	44.6–55.1	

## Data Availability

Data are available from the corresponding author upon reasonable request.
